# Location-Allocation Model to Improve the Distribution of COVID-19 Vaccine Centers in Jeddah City, Saudi Arabia

**DOI:** 10.3390/ijerph19148755

**Published:** 2022-07-19

**Authors:** Areej Alhothali, Budoor Alwated, Kamil Faisal, Sultanah Alshammari, Reem Alotaibi, Nusaybah Alghanmi, Omaimah Bamasag, Manal Bin Yamin

**Affiliations:** 1 Department of Computer Science, Faculty of Computing and Information Technology, King Abdulaziz University, Jeddah 21589, Saudi Arabia; sshammari@kau.edu.sa (S.A.); obamasek@kau.edu.sa (O.B.); 2 Department of Information System, Faculty of Computing and Information Technology, King Abdulaziz University, Jeddah 21589, Saudi Arabia; 3 Geomatics Department, Faculty of Architecture and Planning, King Abdulaziz University, Jeddah 21589, Saudi Arabia; kfaisal@kau.edu.sa; 4 Center of Excellence in Smart Environment Research, King Abdulaziz University, Jeddah 21589, Saudi Arabia; 5 Department of Information Technology, Faculty of Computing and Information Technology, King Abdulaziz University, Jeddah 21589, Saudi Arabia; ralotibi@kau.edu.sa (R.A.); nayyadahalghanmi@stu.kau.edu.sa (N.A.); 6 Planning and Transformation Department, Ministry of Health, Jeddah 21176, Saudi Arabia; mabinyamin@moh.gov.sa

**Keywords:** location-allocation, geographic information system, point of dispensing, network analysis, COVID-19, vaccine centers

## Abstract

The correct distribution of service facilities can help keep fixed and overhead costs low while increasing accessibility. When an appropriate location is chosen, public-sector facilities, such as COVID-19 centers, can save lives faster and provide high-quality service to the community at a low cost. The purpose of the research is to highlight the issues related to the location of COVID-19 vaccine centers in the city of Jeddah, Saudi Arabia. In particular, this paper aims to analyze the accessibility of COVID-19 vaccine centers in Jeddah city using maximal coverage location problems with and without constraint on the number and capacity of facilities. A maximal coverage model is first used to analyze the COVID-19 vaccination coverage of Jeddah districts with no restriction on the facility capacity. Then, a maximize capacitated coverage method is utilized to assess the centers’ distribution and demand coverage with capacity constraints. Finally, the minimize facilities model is used to identify the most optimal location required to satisfy all demand points with the least number of facilities. The optimization approaches consider the objective function of minimizing the overall transportation time and travel distance to reduce wastage on the service rate provided to the patients. The optimization model is applied to a real-world case study in the context of the COVID-19 vaccination center in Jeddah. The results of this study provide valuable information that can help decision-makers locate and relocate COVID-19 centers more effectively under different constraints conditions.

## 1. Introduction

Public health emergencies, either naturally occurring or man-made, are adverse events that pose an urgent threat to the public’s health. To effectively mitigate, prevent, or treat the effects of public health emergencies and to ensure public safety, prior preparation and planning for immediate response are required. Planning for a rapid response includes locating point of dispensing (POD) sites to aid the distribution and dispensing of medical countermeasures such as a vaccine and antibiotics [1]. PODs are used to increase the capacity of providing health services and lessen the burden of accessing public medical institutions during crises, vaccination campaigns, and mass testing. The purpose of establishing PODs and the associated spatial and temporal constraints make their location assignment a challenging and critical task, especially during public health emergencies [2]. To choose the proper number and sites for PODs, various variables need to be gathered, including population distribution, road network data, public transit data, distribution of businesses, households, health centers, and other public facilities [3]. These variables are assessed using mathematical location modeling techniques and geographic information systems (GIS) to choose the optimal PODs while maximizing public access. The candidate locations for emergency mass dispensing can be any public facilities that accommodate a large number of individuals with high-capacity parking spaces, such as convention centers, major sporting arenas, public buildings, and in some cases, schools or shopping centers.

Choosing the optimal number and location of PODs for effective mass dispensing in public health emergencies is a location-allocation problem [4]. A location-allocation model is a strategic decision-making problem that aims to identify the optimal locations for facilities that entail concurrently identifying a set of facility locations and assigning spatially distributed sets of demands to these facilities with the aim of improving the existing distribution [5]. The location and allocation of PODs should be efficient in order to supply the required services to a large number of the targeted population. Researchers have generally adopted one of the two methodologies to solve healthcare facility location-allocation problems, namely the deterministic approach and the uncertain approach. In the deterministic approach, location-allocation problems are formulated as a mathematical (linear) model that optimizes single or multiple health policy objectives, such as minimizing travel time/ distance between demand points and facilities or maximizing demand coverage [6,7]. The uncertain approach is based on the assumption that some of the parameters or the system’s inputs are incomplete or imperfect [8,9,10,11,12]. Additionally, the meta-heuristic methodology was used to solve complex multiple objectives problems, such as minimizing the cost of constructions and maximizing service coverage [13,14].

**Figure 1:**
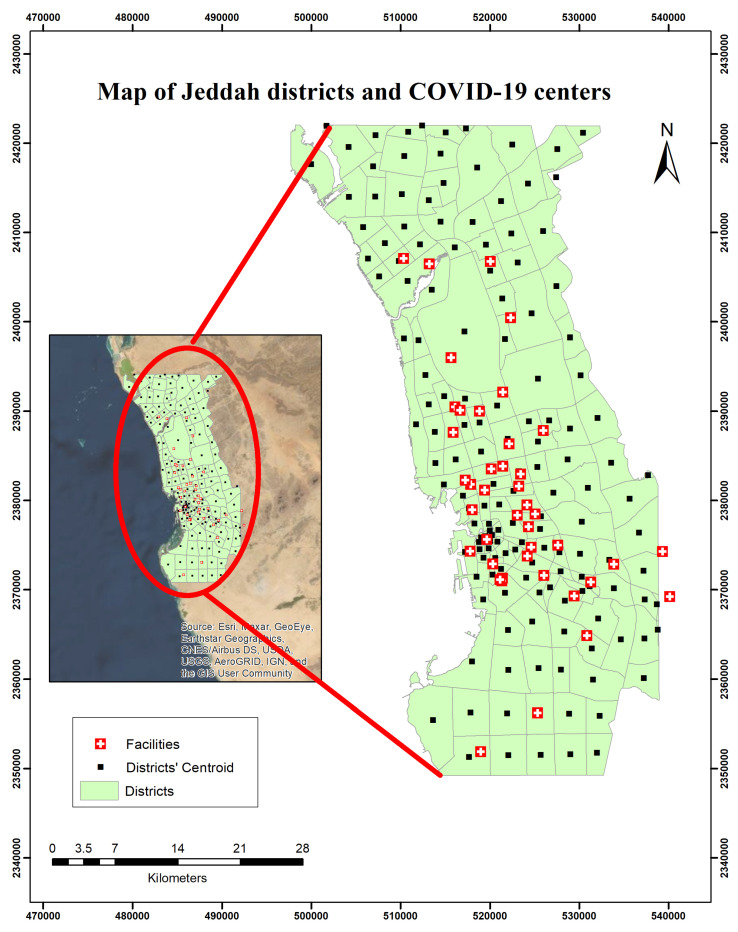
Map of the distribution of existing COVID-19 centers among districts at Jeddah city.

One of the most significant public health emergencies in recent years is the highly infectious coronavirus disease 2019 (COVID-19). COVID-19 is caused by severe acute respiratory syndrome coronavirus 2 (SARS-CoV-2) [15]. The World Health Organization (WHO) declared an influx of public health emergencies of international concern on 30 January 2020 [16]. COVID-19 has triggered a global public health emergency, affecting more than 200 countries and territories. According to WHO [17], the COVID-19 pandemic had more than 260 million confirmed cases and more than 5.2 million reported deaths as of 1 December 2021. In Saudi Arabia, more than 50 thousand COVID-19 cases were confirmed from January 2020 to December 2021, and more than 45 million vaccination doses have been administrated [17]. During the COVID-19 pandemic, most countries have implemented a variety of protective measures that prove to be effective such as social distancing and quarantining [18]. Vaccination is thought to be the most effective technique for averting the pandemic and avoiding the disease’s implications [19]. COVID-19 vaccination centers’ location allocation is concerned with situating these facilities among potential zones in order to deliver efficient and effective services over a large population with spatially distributed demands [20]. As a result, an important and critical issue is how to efficiently distribute vaccine centers so that they are easily accessible to a wide range of the population. Figure 1 demonstrates the current distribution of COVID-19 centers among districts in Jeddah city.

This research analyzes the spatial accessibility of COVID-19 vaccination centers in Jeddah in order to evaluate the locational distribution of COVID-19 facilities under different access and resource constraints. The access to the COVID-19 centers in Jeddah city is evaluated based on the travel distance and transportation time. We selected the maximal covering location problem (MCLP) in analyzing the current distribution of COVID-19 centers in Jeddah. The MCLP is one of several alternative location-allocation models that aims to maximize demand coverage with distance or time constraints. We utilized the maximal coverage model with different impedance cutoffs, which is the maximum travel time required to travel from a demand point to facilities, to assess the accessibility of current COVID-19 centers. The MCLP is thought to be effective when a limited number of facilities are available to cover a large number of demand points, particularly in cases of health emergency location-allocation problems [21].

The maximal coverage is used to analyze COVID-19 vaccination centers’ locations with and without restriction on the facility capacity. First, the maximize coverage model is utilized to locate optimal vaccine sites that maximize demand coverage without constraint on the number of facilities or their capacity. Second, the maximize capacitated coverage model was used to ensure demand coverage while maintaining the restriction of the facility capacity. The model was implemented with different capacity assumptions and impedance cutoffs. Third, the minimize facility problem is used to identify the minimum number of facilities needed to cover demand points, which will help when the number of facilities needs to be reduced. The optimization of demand coverage focuses in this research on the count of the districts rather than the population due to the unavailability of recent census data for the targeted case study.

This helps determine the optimal POD locations to maximize demand coverage while maintaining facility capacity when required and promoting access equity. The precise question can be: Where should the facilities be located in order to maintain a proper distribution of facilities to cover the majority of the population’s needs? In particular, we aim to find answers to the following research questions: (1) How accessible are COVID-19 vaccination centers to Jeddah citizens and residents? (2) When facility capacity is a concern, what is the optimal distribution of facility to maximize coverage of demand points? (3) Which facilities are optimal to maximize the demand coverage when the number of facilities is minimized? To answer these issues, spatial accessibility was investigated using location-allocation algorithms based on the distance between residences represented in the districts’ centroid and facility locations.

### Contribution

This research aims to contribute to society by implementing a COVID-19 vaccine centers location-allocation model that improves the efficiency and effectiveness of reaching vaccine centers under different access and resource scenarios. The proposed models can be utilized to improve the planning of COVID-19 center distribution in Jeddah city, and the application of the models could contribute to speeding up the process of reaching the center to get the vaccine. Studies in the field used location-allocation models to optimize the location of various healthcare facilities, including permanent and temporary emergency sites. This study offers an analysis of health emergency location-allocation problems, particularly COVID-19 vaccination centers’ location-allocation problem. Maximize coverage is one of the most commonly used methods for emergency healthcare facility location-allocation problems. The analysis will provide decision-makers with an insight into optimal vaccine distribution based on various scenarios.

This paper is structured as follows: Section 2 presents the literature review, Section 3 presents the location-allocation models and data, Section 4 provides a description of the research experiment and results, and Section 5 presents a summary and discussion of the results. Finally, Section 6 presents the conclusion of the work.

## 2. Literature Review

Facility location allocation is a strategic decision problem that aims to find an optimal location from a set of candidate sites. The area of location-allocation analysis has a wide range of applications that have been thoroughly researched, such as locating health facilities, retail stores, schools, and police stations. For a broad spectrum of corporate and public organizations, facility location selections are crucial in the strategic design of systems. Poorly located facilities or locating an insufficient number of facilities can significantly increase capital and inventory expenses while degrading customer service [22]. The optimal location can be chosen according to problem-related objectives such as construction cost, travel time, and service demand. The optimal location can be defined as continuous values (i.e., facilities are located anywhere in some bounded region) or discrete values (i.e., a predefined candidate location).

The location of a healthcare facility (HCF) is a critical problem as it has a direct influence on healthcare accessibility and individuals’ satisfactions [21]. In the healthcare domain, facility location studies typically focus on two types of health situations non-emergency and emergency. Non-emergency facility locations problems focus on locating optimal sites for primary care facilities, such as hospitals, clinics [23,24,25], blood banks [26,27,28], medical laboratories [29], mobile health units [30], and rehabilitation centers [31], as well as determining their capacities and allocation required resources. Locating emergency facilities is a more complex and challenging task that requires dealing with critical, urgent, and unpredictable situations. Healthcare emergency facilities are either permanent facilities such as off-site public access devices [32,33], emergency centers [34,35,36] or ambulance stations [37,38], or temporary facilities like temporary medical centers [39,40], sample collection points [41,42], or dispensing points [43,44,45].

Studies conducted to determine optimal HCF sites have either applied deterministic approaches considering that all parameters are given [21], stochastic/uncertain approaches assuming that some of the parameters or the system’s inputs are incomplete or inaccurate [8,45], or hybrid approaches integrating uncertainty in strategic policy modeling [10]. Research in the deterministic paradigm often optimizes a health policy objective using optimization methodologies such as p-median or p-center. P-median problems aim to reduce the total necessary travel distance or time to facilities, which is a prominent indicator of the efficiency [6,46,47]. The p-center, known as the min-max problem, aims to minimize the maximal distance from demand points to their nearest facilities [7]. Another objective employed in the location set and maximal coverage location problems is to maximize population coverage with the minimum number of stations. Depending on the application, problem-specific objectives can be used. For example, in the maximal survival location problems in ambulances, predicted patient survival can be used as a measure of the quality of facility placements [48]. Other researchers have adopted a multiple objectives optimization to choose locations based on a number of goals, such as minimizing the cost of constructions and maximizing service coverage, which gives a more realistic and representative problem formulation of the actual HCF locations decision-making mechanism [24,25]. To efficiently solve complex multiple objectives problems, studies have utilized metaheuristic algorithms to find an optimal or nearly optimal solution in a short time, such as genetic algorithm [13], Lagrangian relaxation [49], simulated annealing [50,51], and particle swarm optimization [12,52].

Critical health emergency situations such as biological attacks, pandemics, or infectious disease outbreaks require a fast and efficient and large-scale dispensing of important medical supplies, prophylaxis, or vaccines to prevent the spread of infectious diseases. Due to the importance of the POD locations, researchers have also used mathematical programming techniques such as linear programming or metaheuristics techniques (e.g., genetic algorithm) to find a POD optimal location based on some simple or complex health policy objectives (e.g., travel and waiting times) [44,53].

Due to the COVID-19 pandemic, several researchers have investigated methods for locating COVID-19 emergency health centers. Manupati et al. [54] proposed a mixed-integer linear programming (MILP) model to develop a plasma supply chain network that simplifies the location of plasma banks as well as the allocation of delivery facilities to these plasma banks for COVID-19. Zhou et al. [55] investigated the inherent spatial variability of COVID-19 transmission in the community and developed vaccine delivery tactics that took spatial prioritizing into account. They suggested a combined agent-based model and SEIR spatial model (susceptible-exposed-infected-recovered) [56] to assess COVID-19 intra-city transmission’s spatial process.

Tavana et al. [57] presented a mixed-integer linear programming model for fair and equitable COVID-19 vaccination distribution in developing countries. The model also considers time-dependent capacity and triple refrigeration requirements (i.e., cold, very cold, and ultra-cold). Lusiantoro et al. [58] proposed a mathematical maximal coverage model to optimize the location of COVID-19 vaccination centers by maximizing the covered demand population and minimizing the total distance traveled by vaccine recipients. The model was demonstrated in a case study of a healthcare center in Yogyakarta, Indonesia. Kuvvetli [59] developed a goal programming model for the location-allocation problem to optimize sample test locations to minimize total distance while maintaining the maximum availability and the minimum number of test sampling centers. The model was applied to two cities in Turkey. Faisal et al. [60] presented a spatial analysis of COVID-19 centers in Jeddah city using kernel density estimation, incremental spatial autocorrelation, and hotspot analysis based on the population distribution and districts map. In this research, we assess the accessibility of COVID-19 vaccination centers using location-allocation maximal coverage under different capacity and access constraints and assumptions.

## 3. Materials and Methods

### 3.1. Location-Allocation Models

The location-allocation problem aims to allocate the COVID-19 vaccine facilities to the closest centroid point of the districts. The focus of this study is to evaluate the optimality of current facilities in Jeddah city and determine the optimal location-allocation decisions using the MCLP method. MCLP seeks to identify the optimal facility locations on network nodes so that demand coverage within a pre-specified period of time or distance is maximized [61]. When there are a small number of facilities are available to cover a large number of demand points, this model is considered promising, especially for health emergency location-allocation problems [21]. In terms of facility selection, unlike the location set coverage problem model, the MCLP model does not aim to reduce the number of facilities required to cover all demand points over a certain distance or time [62]. The MCLP model, on the other hand, provides solutions that span the broadest demand range while adhering to specified distance or time restrictions between supply and demand points. Both the demand and facility locations’ coordinates must be identified with a threshold level of accuracy for the MCLP to work.

The demand and facility points’ coordinates (Table 1 and Table 2) were retrieved from the Ministry of Health Google Earth satellite image. The XY coordinates system is a projected coordinate system with UTM and WGS 1984 for the northern hemisphere and zone 37 N. The number of district centroids at different drive time thresholds of 20, 30, 40, 50, 60, and 120 min to the nearest facilities was evaluated using the Network Analysis extension of the ArcMap system (https://desktop.arcgis.com/, accessed on 15 July 2022) to reflect the current baseline travel time situation in Jeddah. The network analysis tool uses Dijkstra’s algorithm [63] to find the shortest paths. We used MCLP with three conditions and decision-making variables: (1) locating uncapacitated facilities to maximize demand coverage, (2) locating capacitated facilities to maximize demand coverage, and (3) locating uncapacitated facilities while simultaneously maximizing coverage and minimizing the number of facilities. The location-allocation solver in Esri’s ArcGIS is used to solve the three problems using maximize coverage, maximize capacitated coverage, and minimize facilities models. The solver is based on a combination of a Hillsman editing [64], a vertex substitution heuristic [65], and a metaheuristic model for solution refinement. In the following subsections, the general mathematical formulation for MCLP and the capacitated MCLP are provided.

### 3.2. Maximal Covering Location Problem

Based on a network of links and nodes, where nodes are the demands (*n* districts) that need to served by facilities (*p* facilities), $d_{ij}$ is the travel cost/distance associate with the link of two nodes *i* and *j*, and *D* is a pre-defined maximum distance. The MCLP formulated based on Church and ReVelle [66] as follows:

(1a) $$\begin{array}{cccc} & \mathrm{Maximize} & & \sum_{i\in I}h_{i}Y_{i},\; \end{array}$$

(1b) $$\begin{array}{cccc} & \mathrm{subject}\;\mathrm{to} & & \sum_{j\in N_{i}}X_{j}-Y_{i}\geq 0,\;\;\forall i\in I,\; \end{array}$$

(1c) $$\begin{array}{cccc} & & & \sum_{j\in J}X_{j}\leq p,\; \end{array}$$

(1d) $$\begin{array}{cccc} & & & Y_{i}\in \left\{0,1\right\},\;\;\forall i\in I, \end{array}$$

(1e) $$\begin{array}{cccc} & & & X_{j}\in \left\{0,1\right\},\;\;\forall j\in J, \end{array}$$

where:

I= the set of demand nodes {1,..,i,..,m},
J= the set of potential facilities {1,..,j,..n},
D= a pre–defined maximum distance,
$d_{ij}$ = the distance from a demand *i* to facility *j*,
$h_{i}$ = the number of demands at a given node *i* (e.g., the population at a given node),
$N_{i}$ = the set of facilities nodes *j* that can cover demand node *i*. $N_{i}=\left\{j|d_{ij}\leq D\right\}$,
$X_{j}$ = a binary variable indicates whether a facility is located at *j* (1) or not (0),
$Y_{i}$ = a binary decision variable indicates whether the node/demand *i* is covered (1) or not (0).

The objective function, (1a), maximizes the number of covered demands by facilities. Constraint (1b) ensures that demand will be only considered covered if a facility is located within a *D* distance from the demand point. Constraint (1c) ensures that the number of located facilities is less than or equal to the total number of potential sites. Constraints (1d) and (1e), are integrality constraints.

To consider facility capacity in the MCLP problem, the earliest approaches in the field [67,68] have added the capacity constraints into the mathematical formulation of MCLP to ensure that the demand allocated to facilities will not exceed the maximum capacity of the facility. Given the notational convention suggested above, the capacitated maximal coverage problem is formulated based on Current and Storbeck [67] as follows:

(2a) $$\begin{array}{cccc} & \mathrm{Minimize} & & \sum_{i\in I}h_{i}U_{i},\; \end{array}$$

(2b) $$\begin{array}{cccc} & \mathrm{subject}\;\mathrm{to} & & \sum_{j\in N_{i}}W_{ij}+U_{i}=1,\;\;\forall i\in I,\; \end{array}$$

(2c) $$\begin{array}{cccc} & & & \sum_{i\in M_{j}}h_{i}W_{ij}-k_{j}X_{j}\leq 0,\;\forall j\in J, \end{array}$$

(2d) $$\begin{array}{cccc} & & & \sum_{j}X_{j}\leq p,\; \end{array}$$

(2e) $$\begin{array}{cccc} & & & X_{j}\in \left\{0,1\right\},\;\;\;\forall j\in J, \end{array}$$

where:

$U_{i}$ = the percentage of uncovered demands,
$W_{ij}$ = the fraction of demand at node *i* assigned to facility *j*,
$k_{j}$ = the capacity of a facility location,
$M_{j}=$ the set of demand nodes *i* that can be covered by a facility node *j*. $M_{j}=\left\{i|d_{ij}\leq D\right\}$.

The objective function in (2a) minimizes the number of uncovered demands. Constraint (2b) ensures that demand will be considered covered only if the travel time/ distance between demand *i* and facility *j* is less than the maximum travel time or distance. Constraint (2c) ensures that the total demand assigned to a facility does not exceed its capacity. Constraint (2d) ensures that the number of located facilities is less than or equal to the total number of potential sites. Constraint (1e) is integrality constraint.

#### 3.2.1. Maximize Coverage

To maximize demand coverage, MCLPs were utilized to locate optimal facilities from a set of pre-defined facility locations (COVID-19 vaccination centers) and allocate demands (Jeddah districts) to maximize demand coverage within a given distance or travel time. A demand point is considered covered by a facility if the travel time between the demand point and the facility is less than the pre-defined cost threshold. This threshold is known as the coverage radius or impedance cutoff in ArcGIS. The set of pre-defined facilities and demand points are not required to be equivalent in this problem, and a facility can cover an unlimited number of demand points within the pre-defined distance.

#### 3.2.2. Maximize Capacitated Coverage

The goal of the maximize capacitated coverage (MCC) problem is to locate facilities to cover as much demand as possible while considering the capacity of the facility as a constraint. The capacity of facilities is a predefined numeric value (integer value between 1 and 1000) that indicates the maximum number of demands (districts) allowed to be covered by each facility. Since the actual capacity of vaccination centers is not available, we tested the maximal capacitated coverage approach using different capacity assumptions.

#### 3.2.3. Minimize Facilities

Minimizing facilities problems is similar to the maximum coverage problem, in which the main goal is to choose facilities to serve as many demand points as possible, but the number of facilities is to be minimized.

### 3.3. Study Area

Jeddah is situated on the Red Sea coast in western Saudi Arabia, with a total area of 1686 km${}^{2}$ and longitude E 39${}^{\circ }$11,052.6900 and latitude N 21${}^{\circ }$32,032.5700. Jeddah is the kingdom’s second-biggest metropolis, behind Riyadh, and the main city in Makkah Province. Jeddah’s overall population is above 4 million, with an annual growth rate of 2.3 percent.

### 3.4. Data

The dataset is obtained from collecting information regarding all districts of Jeddah city and the number of COVID-19 vaccine centers available in the city. We gathered feature datasets from the topographic map of Jeddah, such as road lines, administrative boundaries, COVID-19 centers, and settlement layers obtained from the Saudi ministry of health. Demand points were represented by the centroid of the districts. The geometric center, often known as the centroid, is a point that represents vector data (multipoint, line, and area features). Figure 1 presents the distribution of existing COVID-19 centers among districts at Jeddah city.

### 3.5. Objective Function

The objectives are to determine the optimal allocation of COVID-19 vaccine facilities to maximize demand coverage, to find the optimal distribution of the facilities under different capacity constraints, and to find the optimal location-allocation distribution of COVID-19 vaccine that simultaneously maximizes demand coverage and minimizes facilities.

### 3.6. Software

In the location and allocation studies of the COVID-19 facilities, geographic information systems (GIS), linear programming methods, and mathematical models are used. The GIS feature of network analysis has been extensively employed to tackle location-allocation problems. GIS allows for the collection and analysis of locational data, making it useful for determining facilities’ locations. The shortest paths or the service areas of the facilities can be determined using network analysis [69]. The majority of our maps in this study were created utilizing the Network Analyst tool extension in ArcMap version 10.8.1 to analyze data. We used ArcMap to perform the network analysis of the district, street maps, and COVID-19 centers in Jeddah. ArcMap software is developed by Environmental Systems Research Institute (Esri), Redlands, CA, USA.

## 4. Experiments and Results

### 4.1. Maximize Coverage

The maximize coverage method is used to select facilities such that all or most of the demand points would have access to facilities within a specified impedance cutoff. The district centroids represent the demand nodes, and the facilities are distributed based on their current locations in Jeddah city. The total number of COVID-19 centers available in Jeddah city is 42 centers and the number of the demand points is 156 districts. We used the location-allocation problem to maximize the coverage of the currently available facilities. The Euclidean distance between the facilities and the demand nodes was used to define the objective function of minimizing the travel time. The following assumptions have been used prior to the implementation:

- Facilities are uncapacitated.
- The impedance is based on time in minutes.
- The travel type is from demand point to facility.
- The impedance transformation is linear.
- Roads are bidirectional.

**Figure 2:**
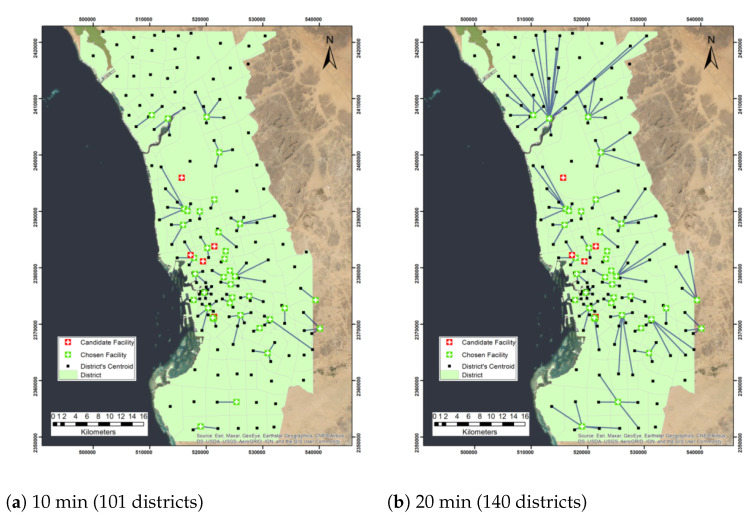

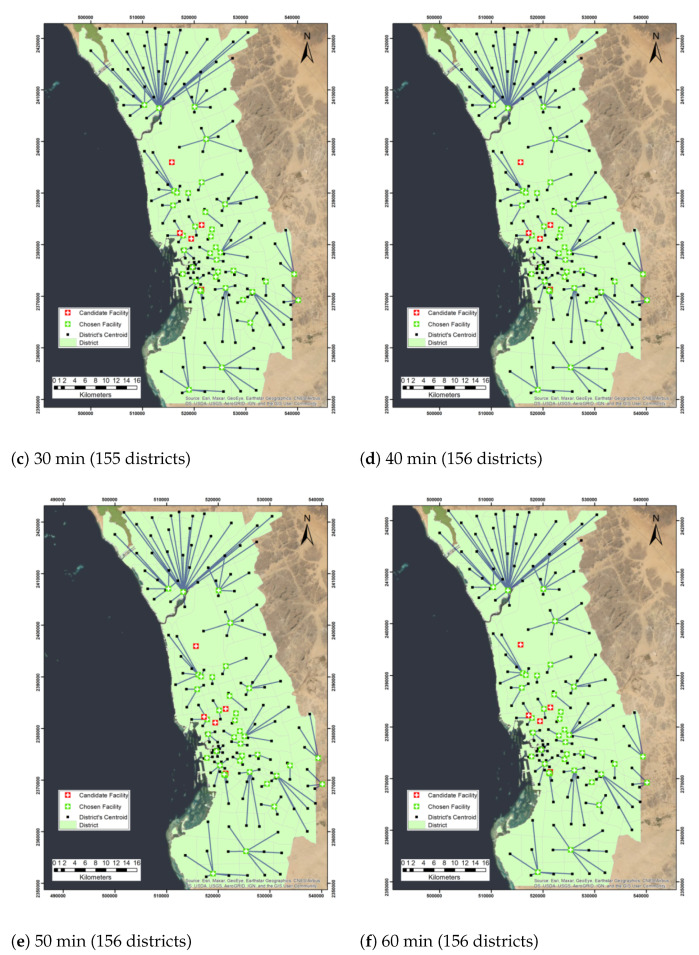
Network analysis map of the maximum coverage problem with different cutoff impedance times.

Different impedance cutoffs (times) are used as a preliminary evaluation of the current distribution of the facilities with regard to the centroid districts. The impedance cutoffs used are 10, 20, 30, 40, 50, and 60 min. We tested the location-allocation approach with different impedance cutoffs to evaluate the accessibility of the centers under different travel time constraints and determine the most accessible centers to districts. This will help decision-makers choose the most accessible centers when the number of facilities needs to be reduced. We first used the 10 min impedance cutoff, and we found that 37 facilities were chosen and 5 candidate facilities were not allocated, which are King Abdulaziz Airport, King Abdulaziz Hospital, King Fahad General Hospital, Al Aziziyah Maternity and Children Hospital, and Al Mishrifah Health Center (Table 6). Moreover, 101 districts can have access to facilities within 10 min. It was found when the impedance cutoff was 20 min, 140 districts out of 156 districts were able to reach facilities, and for a 30 min impedance cutoff, almost all districts can reach facilities (155 districts) except for one district, which is the Al-Rabie district. Moreover, within the 40 impedance cutoff, all the 156 Jeddah districts can have access to the 37 chosen facilities out of 42 facilities. Figure 2 presents the network analysis maps of the maximum coverage problem with different impedance cutoffs. This analysis shows that the current network of facilities was more than sufficient in covering the population districts if a 40 min impedance cutoff is assumed. Table 3 presents the number of located demands and allocated facilities using the maximize coverage problem.

### 4.2. Maximize Capacitated Coverage

The maximize capacitated coverage is used in location allocation when facilities have limited capacity, such that all or the largest amount of demand may be satisfied without any facility exceeding its capacity. Furthermore, this strategy selects the set of facilities to minimize the entire sum of weighted impedance (demand assigned to a facility multiplied by impedance to or from the facility). Similar to the maximize coverage problem, the following assumptions are made in solving this model:

- Each facility has a limited capacity to cover demand from a fixed number of districts (analyzed scenarios with three and four districts).
- The impedance is based on time in minutes.
- The travel type is from demand point to facility.
- The impedance transformation is linear (equal to the cost of the shortest path between the demand point and the facility).
- Roads are bidirectional.

When using maximize capacitated coverage problem, we first considered the maximum capacity of three districts with a similar range of impedance cutoffs to the maximize coverage problem. We found all facilities were chosen except three facilities, which are King Abdulaziz Airport, King Abdulaziz Hospital, and Al Aziziyah Maternity and Children Hospital, and 94 districts were covered out of 156 districts when impedance cutoff equal to 10 (Table 6). In addition, with an impedance cutoff of 20 min, we found out that all facilities were chosen except one facility, King Abdulaziz Airport, with a total of 112 districts covered in 20 min. On the other hand, when cutoff times changed to 30, 40, 50, and 60 min, all facilities were chosen to cover 124, 126, 126, and 126 districts, respectively.

**Figure 3:**
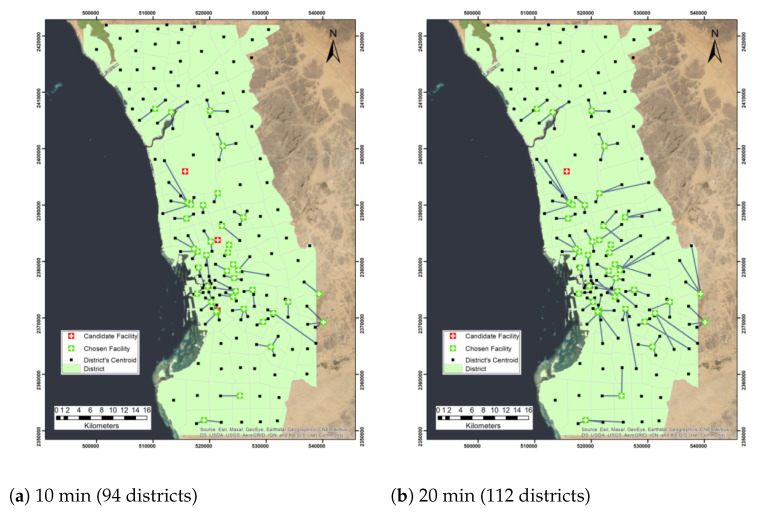

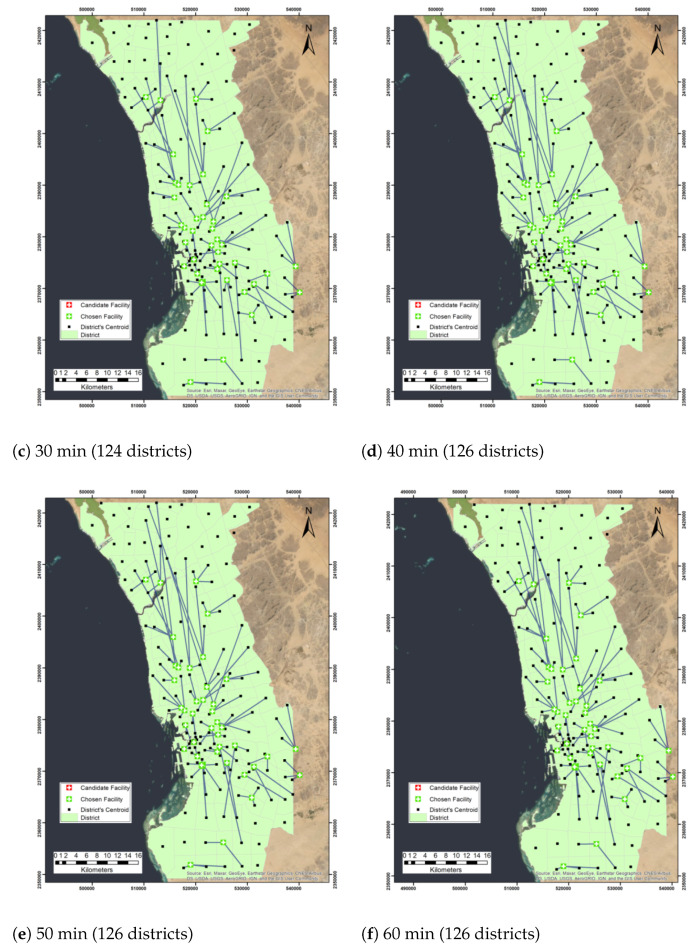
Network analysis map of the maximize capacitated problem with facility capacity of 3 districts and different cutoff impedance times.

**Figure 4:**
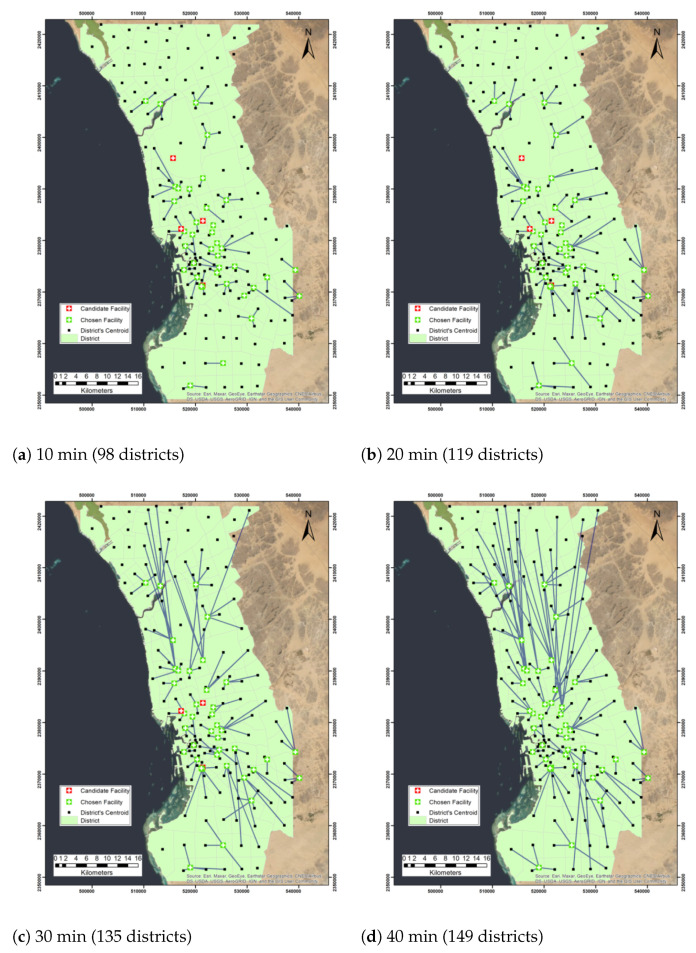

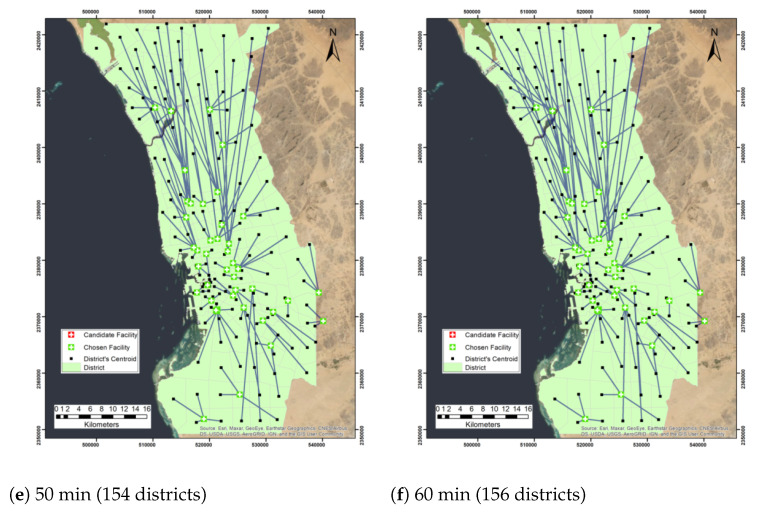
Network analysis map of the maximize capacitated problem with facility capacity of 4 districts and different cutoff impedance times.

Furthermore, we repeated the experiment with a maximum capacity of 4, we found that when the impedance cutoff was 10 min, 98 districts were covered with 38 facilities. All facilities were chosen in the network except four facilities King Abdulaziz Airport, King Abdulaziz Hospital, King Fahad General Hospital, and Al Aziziyah Maternity and Children Hospital (Table 6). On the other hand, when 20, 30, 40, 50, and 60 impedance cutoffs were selected, 119, 135, 149, 154, and 156 districts were covered, respectively. Figure 3 and Figure 4 demonstrate the maximize capacitated coverage problem solution maps under the assumption of facility capacities of three and four districts, respectively. Table 4 shows the number of covered demands and allocated facilities when the maximum capacity of each facility is three districts or four districts.

### 4.3. Minimizing Facilities

The goal of this analysis is to increase coverage while reducing the number of facilities. This approach allows selecting the smallest number of facilities required to meet all or a portion of demand points within a certain impedance cutoff. We applied the minimize facilities problem with different impedance cutoffs. Similar to the previous problems, we use the analysis with the following assumptions:

- Facilities are uncapacitated.
- The impedance is based on time in minutes.
- The travel type is from demand point to facility.
- The impedance transformation is linear.
- Roads are bidirectional.

When analyzing the minimize facilities problem with a 10 min impedance cutoff, we found that 15 facilities were chosen to cover 101 districts. The chosen facilities include the International Medical Center Hospital, King Abdullah Medical Complex, and Obhur Medical Center (see Table 6). Similarly, when we applied the minimize facilities method with an impedance cutoff of 15 min, 10 facilities were chosen out of 42 facilities to connect 122 districts (Table 6).

When selecting an impedance cutoff of 20 min, 9 facilities were chosen out of 42 facilities to cover 140 districts for 20 min. We repeated the same process; however, changing the impedance cutoff to 25 min, we found that 148 districts can have access to 7 chosen facilities which are Obhur Medical Center, Al Thaghr Hospital, Ophthalmology Hospital, Al Majed Medical Center, Al Qwizain Medical Center, Al Harazat Health Center, and Prince Abdullah Al Faisal Stadium.

**Figure 5:**
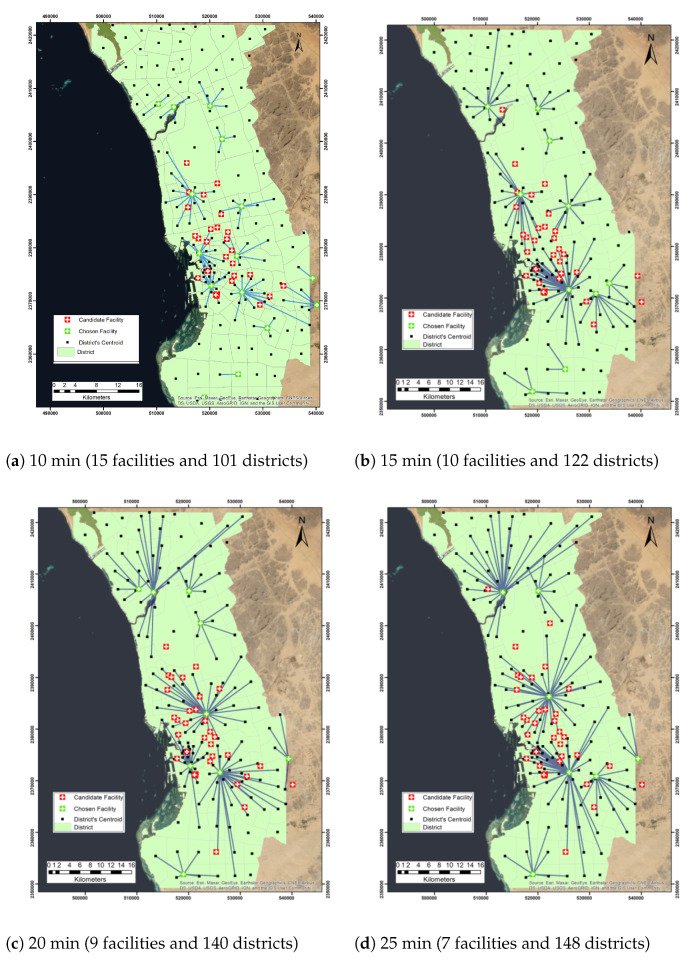

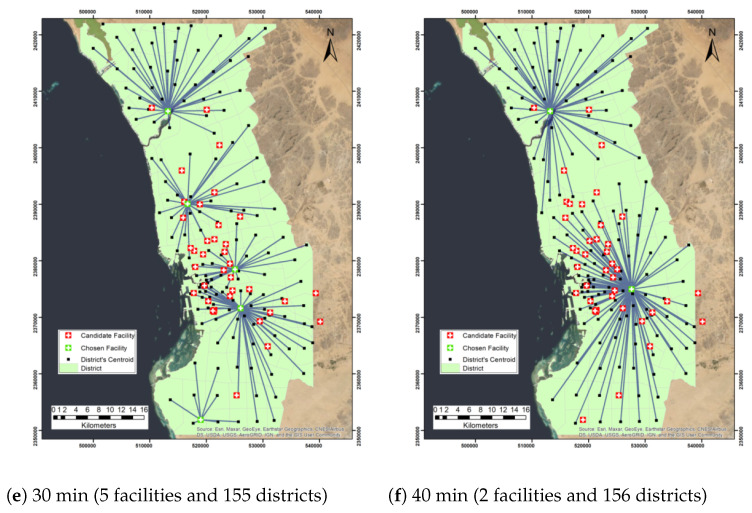
Network analysis map of the minimize facilities problem with different impedance cutoff.

When choosing an impedance cutoff of 30 min with linear impedance transformation, 5 facilities were allocated out of 42 facilities to cover 155 districts. The selected centers are Obhur Medical Center, Health Centre AlBawadi, East Jeddah General Hospital, Al Qwizain Medical Center, and Prince Abdullah Al Faisal Stadium. Selecting an impedance cutoff of 40 min with linear impedance transformation allocates 2 facilities out of 42 facilities to cover 156 districts. The selected centers are Obhur Medical Center and Al Rawabi Health Center. Figure 5 shows the network analysis of the minimize facility problem. Table 5 presents the number of located demands and allocated facilities when using different impedance cutoff.

## 5. Discussion

As shown in Table 2, Table 3, Table 4, Table 5 and Table 6 the result of maximize coverage problem indicates that as we increase the impedance cutoff more districts are covered. These results suggest that it is possible to enhance the coverage of uncovered demand nodes by increasing the impedance cutoff. It can be concluded that improving the coverage of uncovered nodes comes at the expense of increasing the travel time; hence there is a trade-off between maximizing the coverage and reducing the time. Moreover, in the maximize coverage problem, it is noticed that five facilities were not allocated to any demand nodes in all the impedance cutoff, which are King Abdulaziz Airport, King Abdulaziz Hospital, King Fahad General Hospital, Al Aziziyah Maternity and Children Hospital, and Al Mishrifah Health Center. This analysis might interest the decision makers to consider relocating some of the centers to improve the distribution of vaccination centers. On the other hand, the result of the minimize facilities problem highlights that as the impedance cutoff increases, more districts can be covered, and fewer facilities can be chosen as service centers. This can be shown clearly in the result of 40 impedance cutoff time that only two facilities are chosen to cover all Jeddah districts. It is also noticed that in all the impedance cutoff times, the same mentioned five facilities in the maximize coverage problem were still not chosen, which supports the suggestion of considering the optimization of relocating some of the facilities. Comparing the results of maximize capacitated coverage problem, it can be noticed that in a maximum capacity of three, not all facilities can serve all districts even if we increase the impedance cutoff time. Whereas in the maximize capacity of four, as the impedance cutoff time increases, all facilities can be chosen to serve more districts. It is worth mentioning that in the early cutoff time of both maximize capacitated coverage problem of the three and four capacity, three centers of the five previously mentioned facilities in maximize coverage and minimize facilities were still not utilized which are King Abdulaziz Airport, King Abdulaziz Hospital, Al Aziziyah Maternity, and Children Hospital.

The analysis presented in this study provides insight into the spatial distribution of COVID-19 with respect to the districts of Jeddah City; however, a comparison between the analysis conducted in this study and an analysis with the complete data, including recent population census data, deserves further investigation. It is also important to take into account the number of medical staff and available cooling devices when determining and estimating the capacity of vaccination sites. Unfortunately, this data is not currently available but will be considered in future work.

## 6. Conclusions

The geographical accessibility of healthcare resources has long been a topic of interest in public health research. Several studies have looked into analyzing the location and the accessibility of healthcare facilities, including permanent and emergency facilities. Studying and COVID-19 health facilities (vaccination and samples test) locations have attracted many researchers recently. These studies have looked into COVID-19 vaccination distributions sites [57], vaccination centers [58], and sample test sites [59]. These studies were conducted using various location-allocation models and applied to different European and Asian cities. In this research, we evaluate the current location of COVID-19 centers in Jeddah city using different objective functions and constraints conditions. We first examined the accessibility of COVID-19 centers using the maximum covering model. We also assessed the accessibility of the centers with the assumption that each facility is capacitated to cover a limited number of districts. We also locate the optimal centers that maximize the coverage and minimize the number of facilities. This analysis has the potential to aid in the redressing of unequal COVID-19 facilities distribution. The analysis showed the uneven allocation of the existing facilities in some districts compared to other districts in the city, especially when the capacity of COVID-19 centers is considered. This may deter a huge number of people from getting tested and getting COVID-19 vaccines. The results show that if the capacity of COVID-19 centers is not a concern, all demand points can reach vaccination centers in 30–40 min. In the case of capacitated facilities, less number of demand points are covered by vaccination centers. The results also show that when minimizing the number of centers, five centers are chosen to cover the majority of districts (155 districts) within a 30 impedance cutoff.

## Figures and Tables

**Table 1:** A sample of the Jeddah city COVID-19 vaccine facilities with coordinate information.

Facility’s Name	Vaccine Type	x	y
King Abdulaziz Airport	Pfizer	515,688	2,395,991
King Abdulaziz University	Pfizer	524,344	2,377,067
Maternity and Children’s Hospital	Pfizer	517,872	2,381,778
Jeddah field hospital	Pfizer	516,132	2,390,450
Specialized clinics Comprehensive National Guard	Pfizer	523,252	2,381,605
International Medical Center Hospital	Pfizer	518,054	2,378,941
Prince Abdulmajeed PHC Center	AstraZeneca	529,472	2,369,307
King Abdullah Medical Complex	AstraZeneca	510,369	2,407,092
King Abdulaziz Hospital	AstraZeneca	521,435	2,371,302
Al Mahgar Medical Center	AstraZeneca	521,359	2,370,983
Obhur Medical Center	AstraZeneca	513,239	2,406,473
Health Centre AlBawadi	AstraZeneca	516,702	2,390,092

**Table 2:** Sample of Jeddah’s districts and their centroids.

District Name	x Centroid (m)	y Centroid (m)
Al Thagur	523,617	2,375,293
Al Sharafeyyah	519,394	2,379,374
Al Azizeyyah	520,155	2,383,348
Al Dahyh	521,946	2,356,167
Al Naeem	514,888	2,391,636
Al Ajaweed	532,149	2,366,756
Al Khaledeyyah	513,925	2,384,152
Al Basateen	512,025	2,397,900
Al Wazeereyyah	524,073	2,371,332
Al Rabbwah	518,856	2,388,693
Al Faysaleyyah	519,020	2,385,444
Al Ameer Fawaz Al Shamaly	531,195	2,370,382
Al Hada	531,444	2,363,401
Al Ameer Abdoulmajjed	528,407	2,368,760
Al Sahefah	520,266	2,376,112
Al Gharbeyah	512,434	2,421,965
Al Zohor	510,880	2,421,238
Al Hazazia	514,508	2,418,802
Al Eusala	532,077	2,389,199
Betrumeen	520,328	2,371,655
Al Hamadhnyah	520,046	2,405,711
Al Barakah	529,009	2,351,588
Al Mountazahat	530,106	2,374,006
Al Samer	525,395	2,386,573
Al Worood	521,071	2,379,535
Al Bashaer	518,079	2,411,159
Al Sheraa	509,818	2,406,800
Al Ammareyyah	520,031	2,376,598
Al Kandarah	520,956	2,376,690

**Table 3:** Analysis results of the maximize coverage problem with different impedance cutoffs and number of chosen facilities and covered districts.

Impedance Cutoff	No. of Facilities	No. of Districts
10	37	101
20	37	140
30	37	155
40	37	156
50	37	156
60	37	156

**Table 4:** Analysis results of the maximize capacitated coverage problem with different impedance cutoffs and number of chosen facilities and covered districts.

Impedance Cutoff	Capacity = 3 Districts		Capacity = 4 Districts	
	No. of Facilities	No. of Districts	No. of Facilities	No. of Districts
10	39	94	38	98
20	41	112	38	119
30	42	124	39	135
40	42	126	42	149
50	42	126	42	154
60	42	126	42	156

**Table 5:** Analysis results of the minimize facilities problem with different impedance cutoffs and number of chosen facilities and covered districts.

Impedance Cutoff	No. of Facilities	No. of Districts
10	15	101
15	10	122
20	9	140
25	7	148
30	5	155
40	2	156

**Table 6:** A summary of the allocated facilities and a number of located districts using the maximize coverage, minimize facilities, and maximize capacitated coverage (MCC) with different impedance cutoffs.

No.	Facility Name	Minimize Facilities						Maximize Coverage						MCC Capacity 3						MCC Capacity 4					
		10	15	20	25	30	40	10	20	30	40	50	60	10	20	30	40	50	60	10	20	30	40	50	60
1	King Abdulaziz Airport															✓	✓	✓	✓			✓	✓	✓	✓
2	King Abdulaziz University							✓	✓	✓	✓	✓	✓	✓	✓	✓	✓	✓	✓	✓	✓	✓	✓	✓	✓
3	Maternity and Children’s Hospital							✓	✓	✓	✓	✓	✓	✓	✓	✓	✓	✓	✓	✓	✓	✓	✓	✓	✓
4	Jeddah field hospital							✓	✓	✓	✓	✓	✓	✓	✓	✓	✓	✓	✓	✓	✓	✓	✓	✓	✓
5	Comprehensive National Guard							✓	✓	✓	✓	✓	✓	✓	✓	✓	✓	✓	✓	✓	✓	✓	✓	✓	✓
6	International Medical Center	✓						✓	✓	✓	✓	✓	✓	✓	✓	✓	✓	✓	✓	✓	✓	✓	✓	✓	✓
7	Prince Abdulmajeed PHC Center							✓	✓	✓	✓	✓	✓	✓	✓	✓	✓	✓	✓	✓	✓	✓	✓	✓	✓
8	King Abdullah Medical Complex	✓	✓	✓				✓	✓	✓	✓	✓	✓	✓	✓	✓	✓	✓	✓	✓	✓	✓	✓	✓	✓
9	King Abdulaziz Hospital														✓	✓	✓	✓	✓				✓	✓	✓
10	Al Mahgar Medical Center							✓	✓	✓	✓	✓	✓	✓	✓	✓	✓	✓	✓	✓	✓	✓	✓	✓	✓
11	Obhur Medical Center	✓		✓	✓	✓	✓	✓	✓	✓	✓	✓	✓	✓	✓	✓	✓	✓	✓	✓	✓	✓	✓	✓	✓
12	Health Centre AlBawadi	✓	✓			✓		✓	✓	✓	✓	✓	✓	✓	✓	✓	✓	✓	✓	✓	✓	✓	✓	✓	✓
13	Al Thaghr Hospital		✓		✓			✓	✓	✓	✓	✓	✓	✓	✓	✓	✓	✓	✓	✓	✓	✓	✓	✓	✓
14	Ophthalmology Hospital				✓			✓	✓	✓	✓	✓	✓	✓	✓	✓	✓	✓	✓	✓	✓	✓	✓	✓	✓
15	King Fahad General Hospital													✓	✓	✓	✓	✓	✓				✓	✓	✓
16	East Jeddah General Hospital	✓				✓		✓	✓	✓	✓	✓	✓	✓	✓	✓	✓	✓	✓	✓	✓	✓	✓	✓	✓
17	Mental health hospital in Jeddah							✓	✓	✓	✓	✓	✓	✓	✓	✓	✓	✓	✓	✓	✓	✓	✓	✓	✓
18	Al Majed Medical Center	✓	✓	✓	✓			✓	✓	✓	✓	✓	✓	✓	✓	✓	✓	✓	✓	✓	✓	✓	✓	✓	✓
19	University District PHC Center							✓	✓	✓	✓	✓	✓	✓	✓	✓	✓	✓	✓	✓	✓	✓	✓	✓	✓
20	Al Aziziyah M and C Hospital														✓	✓	✓	✓	✓				✓	✓	✓
21	Tayba Medical Center	✓						✓	✓	✓	✓	✓	✓	✓	✓	✓	✓	✓	✓	✓	✓	✓	✓	✓	✓
22	Al Rayan Medical Center	✓	✓	✓				✓	✓	✓	✓	✓	✓	✓	✓	✓	✓	✓	✓	✓	✓	✓	✓	✓	✓
23	Madain Al Fahd Medical Center							✓	✓	✓	✓	✓	✓	✓	✓	✓	✓	✓	✓	✓	✓	✓	✓	✓	✓
24	Al Balad Medical Center							✓	✓	✓	✓	✓	✓	✓	✓	✓	✓	✓	✓	✓	✓	✓	✓	✓	✓
25	Al Thaalba Medical Center	✓		✓				✓	✓	✓	✓	✓	✓	✓	✓	✓	✓	✓	✓	✓	✓	✓	✓	✓	✓
26	Al Qryniah Medical Center	✓	✓					✓	✓	✓	✓	✓	✓	✓	✓	✓	✓	✓	✓	✓	✓	✓	✓	✓	✓
27	Al Qwizain Medical Center	✓	✓	✓	✓	✓		✓	✓	✓	✓	✓	✓	✓	✓	✓	✓	✓	✓	✓	✓	✓	✓	✓	✓
28	Al Rabwah PHC Center							✓	✓	✓	✓	✓	✓	✓	✓	✓	✓	✓	✓	✓	✓	✓	✓	✓	✓
29	Al Azziziyah Health Center							✓	✓	✓	✓	✓	✓	✓	✓	✓	✓	✓	✓	✓	✓	✓	✓	✓	✓
30	Al Mishrifah Health Center													✓	✓	✓	✓	✓	✓	✓	✓	✓	✓	✓	✓
31	Al Salamah Health Center							✓	✓	✓	✓	✓	✓	✓	✓	✓	✓	✓	✓	✓	✓	✓	✓	✓	✓
32	Al Marwah Health Center							✓	✓	✓	✓	✓	✓	✓	✓	✓	✓	✓	✓	✓	✓	✓	✓	✓	✓
33	Health Center East of The Highway	✓	✓					✓	✓	✓	✓	✓	✓	✓	✓	✓	✓	✓	✓	✓	✓	✓	✓	✓	✓
34	Al Rawabi Health Center						✓	✓	✓	✓	✓	✓	✓	✓	✓	✓	✓	✓	✓	✓	✓	✓	✓	✓	✓
35	Al Rehab Health Center			✓				✓	✓	✓	✓	✓	✓	✓	✓	✓	✓	✓	✓	✓	✓	✓	✓	✓	✓
36	Al Harazat Health Center	✓		✓	✓			✓	✓	✓	✓	✓	✓	✓	✓	✓	✓	✓	✓	✓	✓	✓	✓	✓	✓
37	Kilo 14 Health Center		✓					✓	✓	✓	✓	✓	✓	✓	✓	✓	✓	✓	✓	✓	✓	✓	✓	✓	✓
38	Old Airport Health Center							✓	✓	✓	✓	✓	✓	✓	✓	✓	✓	✓	✓	✓	✓	✓	✓	✓	✓
39	Umm Al Salam Health Center	✓						✓	✓	✓	✓	✓	✓	✓	✓	✓	✓	✓	✓	✓	✓	✓	✓	✓	✓
40	Health Surveillance Center JIP							✓	✓	✓	✓	✓	✓	✓	✓	✓	✓	✓	✓	✓	✓	✓	✓	✓	✓
41	Al Salaam Mall							✓	✓	✓	✓	✓	✓	✓	✓	✓	✓	✓	✓	✓	✓	✓	✓	✓	✓
42	Prince Abdullah Al Faisal Stadium	✓	✓	✓	✓	✓		✓	✓	✓	✓	✓	✓	✓	✓	✓	✓	✓	✓	✓	✓	✓	✓	✓	✓

## Data Availability

Not applicable.
